# Immune checkpoint inhibitor-related diabetes mellitus associated with high signal intensity in diffusion-weighted magnetic resonance imaging of the pancreas at an early clinical stage

**DOI:** 10.1007/s42000-025-00629-3

**Published:** 2025-01-17

**Authors:** Masaki Suzuki, Yushi Hirota, Shin Urai, Masaaki Yamamoto, Keitaro Sofue, Wataru Ogawa

**Affiliations:** 1 Division of Diabetes and Endocrinology, Department of Internal Medicine, Kobe University Graduate School of Medicine, 7-5-2 Kusunoki-cho, Chuo-ku, Kobe, 650-0017 Japan; 2 Division of Diabetes and Endocrinology, Kobe University Hospital, Kobe, Japan; 3 Department of Radiology, Kobe University Graduate School of Medicine, Kobe, Japan

**Keywords:** Nivolumab, Type 1 diabetes, PD-1 antibody, Magnetic resonance imaging

## Abstract

**Introduction:**

Immune checkpoint inhibitors (ICIs) have revolutionized cancer treatment but can give rise to immune-related adverse events such as ICI-related diabetes mellitus (DM).

**Case presentation:**

We herein present the case of a 59-year-old Japanese man with malignant melanoma who developed ICI-related DM after 18 months of nivolumab treatment. He experienced marked hyperglycemia and diabetic ketoacidosis without a personal or family history of diabetes. Laboratory findings revealed initial preservation of insulin secretion but a rapid decline in C-peptide levels in the absence of islet autoantibodies. He was therefore diagnosed with ICI-related DM. This case fulfilled the criteria for fulminant type 1 DM but lacked the typical human leukocyte antigen alleles associated with conventional type 1 diabetes. No metastasis or morphological changes were apparent on CT scans of the pancreas, and magnetic resonance cholangiopancreatography did not show dilation or interruption of the main pancreatic duct. However, diffusion-weighted magnetic resonance imaging revealed high signal intensity with low apparent diffusion coefficient values in the pancreas, likely indicative of fibrosis or infiltration of inflammatory cells.

**Discussion:**

This case underscores that ICI-related DM should be considered a potential immune-related adverse event as well as pointing to the benefit of diffusion-weighted imaging for assessment of pancreatic involvement at an early stage of the disease.

## Introduction

Immune checkpoint inhibitors (ICIs), which include antibodies to cytotoxic T-lymphocyte-associated antigen 4 (CTLA-4), programmed cell death–1 (PD-1), and programmed death-ligand 1 (PD-L1) are now widely administered as anticancer therapeutics. Immune checkpoint molecules inhibit the immune response of T cells, while antibodies to PD-1 relieve such inhibition mediated by PD-1 and its ligand PD-L1 and thereby activate T cell function. However, such promotion of antitumor immunity is associated with the development of immune-related adverse events (irAEs), such as ICI-related diabetes mellitus (DM).

Although irAEs have been identified in various tissues, including the skin, gastrointestinal tract, liver, muscle, nerves, and endocrine organs, such as the thyroid, pituitary, pancreas, and adrenal glands [1], the incidence of ICI-related DM is extremely rare, ranging from 0.9 to 2% [2]. Nevertheless, ICI-related DM is one of the most severe irAEs given that it is accompanied by rapid destruction of pancreatic β cells, leading to the development of diabetic ketoacidosis (DKA) and an insulin-dependent state [3]. It is therefore important that individuals taking ICIs monitor their blood glucose levels regularly during routine clinical care in order to facilitate the early detection or diagnosis of ICI-related DM [2, 4]. Such early detection and diagnosis remain difficult, however, even with regular blood glucose monitoring [5]. In addition, although elevated circulating levels of pancreatic enzymes, such as lipase, have been observed in individuals with ICI-related DM [6, 7], this phenomenon does not consistently serve as a reliable biochemical indicator, underscoring the dearth of diagnostic markers for ICI-related DM other than increased blood glucose levels and emphasizing the need for additional diagnostic approaches.

ICI-related DM, including that induced by antibodies to PD-1, has been found to satisfy the criteria for conventional fulminant or acute-onset type 1 diabetes, as defined by the Japan Diabetes Society [8–10]. Magnetic resonance imaging (MRI) performed early during the onset of fulminant type 1 diabetes has revealed high signal intensity in the pancreas on diffusion-weighted imaging (DWI), which can serve as a diagnostic tool [11]. However, pancreatic MRI features associated with ICI-related DM resembling fulminant type 1 diabetes are not well characterized, particularly at the early clinical stage when accurate diagnosis is crucial.

We herein present the case study of an individual diagnosed with ICI-related DM who developed hyperglycemia and DKA while reporting important pancreatic findings and providing valuable insights into an early stage of the disease.

## Case presentation

A 59-year-old Japanese man with malignant melanoma was treated with the PD-1 antibody nivolumab (3 mg/kg, administered every 2 weeks) for 18 months. He had no personal or family history of diabetes and no signs of pancreatic metastases. His plasma glucose levels remained within normal limits until after he received the 35th dose of nivolumab. At the time of administration of the 36th dose, however, hyperglycemia was observed, with a serum glucose level of 229 mg/dL, whereas his serum C-peptide immunoreactivity (CPR) level was in the normal range at 5.47 ng/mL and his serum amylase concentration was normal. The patient did not show any abdominal symptoms or signs of pancreatitis nor were there any signs of an antecedent infection, prompting monitoring without further evaluation or treatment for hyperglycemia. Eight days after administration of the 36th dose of nivolumab, the patient experienced marked hyperglycemia (552 mg/dL) and metabolic acidosis (pH 7.172, HCO_3_^–^ 6.7 mmol/L, anion gap 27.3 mmol/L) with elevated blood ketone bodies (Table 1). In addition, his hemoglobin A_1c_ level was relatively low (6.8%), indicative of a rapid onset of hyperglycemia. He was therefore diagnosed with DKA and was admitted to the Division of Diabetes and Endocrinology at Kobe University Hospital, Kobe, Japan.

**Table 1:** Clinical data at the onset of DKA or at the time of diagnosis with ICI-related DM

Parameter	Value	Unit	Parameter	Value	Unit
*Urine testing*			*Biochemistry*		
pH	1.028		T-Bil	1.6	mg/dL
Protein	1+		AST	24	U/L
Glucose	4+		ALT	40	U/L
Ketone body	3+		LDH	191	U/L
Blood	1+		γ-GTP	31	U/L
CPR	< 0.1	µg/day	Total protein	8.5	mg/dL
			Albumin	5.2	mg/dL
*Arterial blood gas analysis*			BUN	28.2	mg/dL
pH	7.172		Creatinine	0.95	mg/dL
pO_2_	118.0	mmHg	eGFR	63.7	mL min^–1^ 1.73 m^–2^
pCO_2_	18.9	mmHg	Na	132	mEq/L
HCO_3_^–^	6.7	mmol/L	K	5.3	mEq/L
Base excess	–21.0	mmol/L	Cl	98	mEq/L
Anion gap	27.3	mmol/L	CRP	0.08	mg/dL
			Amylase	14	U/L
*CBC*			Elastase 1	137.4	ng/dL
WBC	9600	/µL	Lipase	20	U/L
Neutrophils	93.0	%	Glucose	552	mg/dL
Lymphocytes	4.0	%	CPR	0.62	ng/mL
Monocytes	3.0	%	IRI	3.5	µU/mL
RBC	5.15	10^6^/µL	HbA_1c_	6.8	%
Hemoglobin	16.0	g/dL	Total ketone body	11,250	µmol/L
Hematocrit	46.3	%	Acetoacetate	2750	µmol/L
Platelets	14.3	10^4^/mL	β-hydroxybutyrate	8500	µmol/L
*Endocrinology*			*Pancreatic islet-related autoantibodies*		
TSH	0.511	µIU/mL	Anti-GAD	< 5.0	U/mL
fT4	1.36	ng/dL	Anti-IA-2	< 0.6	U/mL
Anti-TPO	< 9	IU/mL	Anti-ZnT8	< 10	U/mL
Anti-Tg	< 10	IU/mL	Anti-insulin	< 0.4	%
Anti-TSH receptor	< 0.8	IU/L			

Abbreviations: ICI, immune checkpoint inhibitor; CPR, C-peptide immunoreactivity; CBC, complete blood count; WBC, white blood cell; RBC, red blood cell; TSH, thyroid-stimulating hormone; fT4, free thyroxine; TPO, thyroid peroxidase; Tg, thyroglobulin; T-Bil, total bilirubin; AST, aspartate aminotransferase; ALT, alanine aminotransferase; LDH, lactate dehydrogenase; γ-GTP, γ-glutamyl transpeptidase; BUN, blood urea nitrogen; eGFR, estimated glomerular filtration rate; CRP, C-reactive protein; IRI, immunoreactive insulin; HbA_1c_, glycosylated hemoglobin; GAD, glutamic acid decarboxylase; IA-2, insulinoma-associated antigen-2; ZnT8, zinc transporter 8

The patient was fully aware and alert. His height, body weight, and body mass index were 176 cm, 74.4 kg, and 24.0 kg/m^2^, respectively. No irregularities were detected on physical examination. Laboratory findings at admission revealed that insulin secretion was somewhat preserved but the 24-h urinary CPR concentration on the 2nd day of hospitalization was < 0.1 µg/day and the fasting serum CPR level was below the limit of detection by the 11th day. Serum levels of pancreatic enzymes were normal at admission. Islet autoantibodies, including those to glutamic acid decarboxylase (GAD) and insulinoma-associated antigen-2 (IA-2), were not detected. In addition, antibodies that combat viruses were assessed at two specific time points (days 2 and 21) to ensure that there was no viral infection, with no increase in titer being detected. The patient was therefore diagnosed with ICI-related DM, which also fulfilled the criteria for conventional fulminant type 1 DM [8]. Human leukocyte antigen (HLA) analysis revealed that he harbored HLA-DRB1*04:06-DQB1*03:02 and HLA-DRB1*04:10-DQB1*04:02 haplotypes, which are not usually associated with the acute-onset or fulminant forms of type 1 DM or with ICI-related DM [12–15].

The patient was discharged from the hospital after a stay of 24 days and received multiple daily insulin injections. Given that the patient’s general condition was stable, nivolumab monotherapy was resumed 34 days following the onset of DKA. However, nivolumab was discontinued due to disease progression after administration of the 51st dose and ipilimumab was subsequently initiated as the alternative therapy (3 mg/kg, administered every 3 weeks). In response to the progression of lymph node metastasis after administration of the 4th dose of ipilimumab, the therapeutic regimen was adjusted to incorporate a combination of encorafenib and binimetinib, which continues to be administered at present. Evaluation of treatment efficacy involves trimonthly CT imaging, which has not revealed any structural abnormalities in the pancreas. Serum amylase levels remain within normal ranges. Concurrent management of diabetes is conducted within our department, with glycemic management demonstrating satisfactory improvement, as indicated by a recent HbA_1c_ level of 6.4%. Development of other irAEs, with the exception of thyroid dysfunction, has not been observed to date (56 months after discharge).

**Fig. 1:**
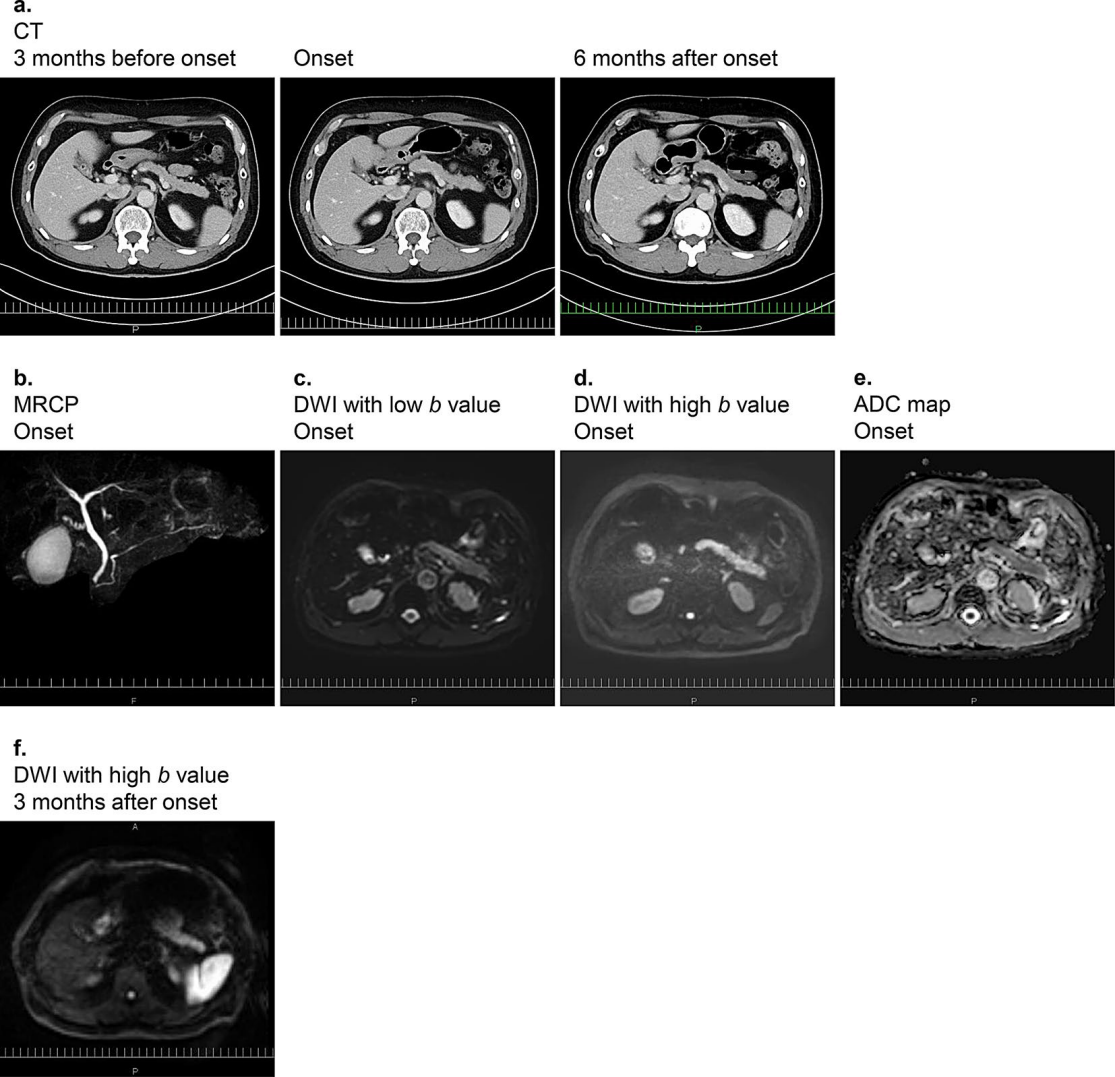
CT and MRI scans of the pancreas. CT scans 3 months before, during, and 6 months after the initial stage of ICI-related DM onset (**a**). A magnetic resonance cholangiopancreatography (MRCP) scan (**b**), DWI scans with low (0 s/mm^2^) (**c**) or high (1000 s/mm^2^) (**d**) *b* values, and an ADC map (**e**) during the initial stage of ICI-related DM onset. No changes in the morphology of the pancreas were apparent on CT, MRCP revealed no enlargement or interruption of the main pancreatic duct, and DWI showed high-intensity signals in the pancreas accompanied by low ADC values. DWI scans with high (1000 s/mm^2^) at 3 months after onset showed a reduction (**f**). Abbreviations: CT, computed tomography; MRCP, magnetic resonance cholangiopancreatography; DWI, diffusion-weighted imaging; ADC, apparent diffusion coefficient; ICI, immune checkpoint inhibitor

We performed a thorough analysis of the images acquired during the initial stage of ICI-related DM onset. Computed tomography (CT) did not reveal metastasis or obvious morphological changes in the pancreas (Fig. 1a) and no clinical progression of malignant melanoma was apparent. In addition, magnetic resonance cholangiopancreatography did not show dilation or interruption of the main pancreatic duct, but did reveal narrowing of the pancreas body and tail (Fig. 1b). DWI with *b =* 0 or a high *b* value (1000 s/mm^2^) revealed no significant enlargement, swelling, or edema but showed high signal intensity in the pancreas (day 2 of hospitalization) (Fig. 1c, d). The apparent diffusion coefficient (ADC) values for the head, body, and tail of the pancreas were 1.294 × 10^–3^, 1.255 × 10^–3^, and 1.135 × 10^–3^ mm^2^/s, respectively, with the mean of these values being 1.228 × 10^–3^ mm^2^/s (Fig. 1e). The ADC values ​​in these regions were lower than those previously reported for nondiabetic individuals [11], indicating that the entire pancreas showed diffusion restriction on DWI. Three months following initial onset of diabetes, MRI scans showed a reduction in diffusion restriction (Fig. 1f).

## Discussion

The case of the patient presented herein highlights the importance of considering ICI-related DM as a potential adverse event of ICI treatment as well as the utility of DWI for assessment of pancreatic involvement in such cases. Individuals diagnosed with ICI-related DM often manifest abnormal results in blood and urine tests that can lead to a definitive diagnosis. However, pancreatic MRI findings in these individuals are rarely reported. Our report therefore provides a highly valuable description of pancreatic MRI results relevant to the treatment of individuals diagnosed with ICI-related DM.

DWI is a diagnostic technique that measures the movement of water molecules within organs, with ADC values calculated based on DWI providing a quantitative assessment of such water movement. Regions of high signal intensity on DWI images correspond to a decrease in ADC values and restricted diffusion, and such decreased ADC values have been found to result from the presence of fibrosis or the infiltration of inflammatory cells [16]. We therefore hypothesize that the regions of high signal intensity revealed by DWI in the pancreas of the present patient are indicative of inflammation and fibrosis.

Recent findings suggest that quantification of the ADC may prove informative for assessment of disease activity in individuals with other autoimmune diseases, such as autoimmune pancreatitis and Crohn’s disease [17, 18]. Pancreatic DWI has also yielded important results in individuals with type 1 DM, which is caused by lymphocytic infiltration of the pancreas and the rapid destruction of pancreatic β cells [11, 19, 20]. In addition, ADC values for the pancreas were found to be substantially lower in individuals with type 1 DM than in nondiabetic control individuals [11]. In the present case, the ADC values for all parts of the pancreas were lower than the previously reported optimal cutoff values for diagnosis of fulminant type 1 DM [11], suggesting that rapid destruction of pancreatic β cells may also occur in ICI-related DM as it does in individuals with fulminant type 1 diabetes.

In the case of pancreatitis associated with ICI treatment, the pancreas typically presents with diffuse or focal enlargement [21]. Pancreatic injury induced by ICIs can lead to reduced endocrine and exocrine pancreatic function and consequent metabolic and nutritional disturbances, and it is often accompanied by abdominal pain, pancreatic enlargement, and elevated circulating levels of pancreatic enzymes [22]. Whereas pancreatic enzyme levels have also been found to be elevated in certain individuals with ICI-related DM [6], atrophy of the pancreas has been detected either at the onset or later in the progression of this disease [23]. ICI-induced acute pancreatitis may also result in pancreatic atrophy [24], however, which can present a challenge to differentiation between ICI-related DM and pancreatitis on the basis of pancreatic morphology.

In the current case, no morphological irregularities of the pancreas were apparent on MRI at the early stage of disease onset, although that does not preclude the eventual development of pancreatic atrophy. The absence of obvious morphological changes in the pancreas together with the fact that the patient did not experience abdominal pain suggests that endocrine function was primarily affected. High signal intensity in DWI of the pancreas may therefore serve as an imaging biomarker reflecting islet damage and inflammatory infiltration at an early stage of ICI-related DM, even in the absence of morphological abnormalities of the pancreas.

Recent pathological findings have revealed increased infiltration of T lymphocytes and macrophages in pancreatic islets, including regions containing α cells and β cells, together with a downregulation of islet PD-L1 expression in individuals with ICI-related DM [25, 26]. The observed destruction of pancreatic β cells in such individuals may be linked to the decrease in PD-L1 expression in these cells resulting from ICI treatment. Further studies are required to investigate the relation between the pancreatic regions of high signal intensity identified by DWI and corresponding pathological features.

A prompt and accurate diagnosis of ICI-related DM is important in individuals treated with ICIs who develop hyperglycemia. However, individuals with type 2 diabetes treated with ICIs or those receiving high-dose glucocorticoid treatment may also experience hyperglycemia [27]. The correct diagnosis of ICI-related DM can therefore be challenging, especially during its early stages or in the absence of symptoms. In such instances, MRI of the pancreas can provide valuable information by detecting the rapid destruction of pancreatic β cells and thereby aid in the early diagnosis of ICI-related DM. Given that the clinical manifestations of ICI-related DM vary widely in terms of rate of onset and timing, additional studies are warranted to evaluate the utility of pancreatic MRI for diagnosis of this condition. Furthermore, it is of importance to investigate the relationship between the regions of high signal intensity revealed by pancreatic DWI and islet damage, including measurement of insulin secretion and its fluctuations over time.

In this study, MRI was not performed prior to the initiation of nivolumab therapy. Moreover, long-term alterations in MRI findings remain uncertain, with the exception of those observed 3 months after the onset of DKA. Hence, further studies are needed to elucidate the association between the initiation of immune processes and specific manifestations observed in MRI and to gain deeper insight into the distinctions between ICI-related DM and conventional fulminant type 1 DM.

Moreover, pancreatic islet autoantibodies were negative and susceptible HLA haplotypes associated with conventional type 1 DM or ICI-related DM were absent in this case. Previous research showed that islet autoantibodies and genotypes susceptible to conventional type 1 DM were negative in 47% and 39% of individuals with ICI-related DM, respectively [28]. In the absence of islet autoantibodies or susceptible HLA, as observed in this case, MRI findings may contribute to the diagnosis of ICI-DM; however, further research is required to elucidate the relationship between autoantibodies or HLA haplotypes and imaging findings.

In conclusion, we here report an individual with ICI-related DM characterized by hyperglycemia and DKA who showed elevated signal intensity with low ADC values in the pancreas on DWI performed at the early stage of disease onset. Quantification of ADC values may therefore prove beneficial for assessment of disease activity in individuals with ICI-related DM. The application of pancreatic MRI may also prove valuable for elucidation of the pathophysiology of hyperglycemia and islet damage in individuals treated with ICIs.

## Data Availability

Data sharing is not applicable to this report as no data sets were generated or analyzed during the current study.
